# Fast Preparation of Porous MnO/C Microspheres as Anode Materials for Lithium-Ion Batteries

**DOI:** 10.3390/nano7060121

**Published:** 2017-05-26

**Authors:** Jing Su, Hao Liang, Xian-Nian Gong, Xiao-Yan Lv, Yun-Fei Long, Yan-Xuan Wen

**Affiliations:** 1 School of Chemistry and Chemical Engineering, Guangxi University, Nanning 530004, China; sujing928@126.com (J.S.); 15253732337@163.com (H.L.); 138786083682050@163.com (X.-N.G.); longyf@gxu.edu.cn (Y.-F.L.); 2 Guangxi Colleges and Universities Key Laboratory of Novel Energy Materials and Related Technology, Nanning 530004, China; 3 Guangxi Novel Battery Materials Research Center of Engineering Technology, Nanning 530004, China; 4 The New Rural Development Research Institute, Guangxi University, Nanning 530004, China; lvxiaoyan666@163.com

**Keywords:** lithium-ion batteries, anode materials, MnO, Co-precipitation, T-shaped microchannel reactor

## Abstract

Porous MnO/C microspheres have been successfully fabricated by a fast co-precipitation method in a T-shaped microchannel reactor. The structures, compositions, and electrochemical performances of the obtained MnO/C microspheres are characterized by X-ray diffraction, field emission scanning electron microscopy (FE-SEM), transmission electron microscopy (HRTEM), Brunauer–Emmett–Teller analysis, charge-discharge testing, cyclic voltammograms, and electrochemical impedance spectra. Experimental results reveal that the as-prepared MnO/C, with a specific surface area of 96.66 m^2^·g^−1^ and average pore size of 24.37 nm, exhibits excellent electrochemical performance, with a discharge capacity of 655.4 mAh·g^−1^ after cycling 50 times at 1 C and capacities of 808.3, 743.7, 642.6, 450.1, and 803.1 mAh·g^−1^ at 0.2, 0.5, 1, 2, and 0.2 C, respectively. Moreover, the controlled method of using a microchannel reactor, which can produce larger specific surface area porous MnO/C with improved cycling performance by shortening lithium-ion diffusion distances, can be easily applied in real production on a large scale.

## 1. Introduction

In the past few decades, rechargeable lithium-ion batteries (LIBs) have attracted considerable attention as a major power source for portable electronic devices and electric vehicles [1,2,3]. However, the commercial anode material, graphite, cannot easily meet the increasing demand for large energy and power densities, with a limited theoretical capacity of 372 mAh·g^−1^ [4,5]. Transition metal oxides have attracted significant research attention due to their two- or threefold improvement in reversible capacity, compared with that of graphite [6,7,8]. Among all of the kinds of transition metal oxides investigated for LIBs, manganese oxide (MnO) has received particular interest because of its high theoretical capacity (755 mAh·g^−1^), relatively low electromotive force (1.032 V vs. Li^+^/Li), high density (5.43 g·cm^−3^), environmental friendliness, and abundance in nature [9,10,11]. However, poor cycling stability and rate capability, owing to the low conductivity and large volume changes during Li-ion insertion/extraction, have hampered the application of MnO [12,13]. Many strategies have been adopted to overcome these shortcomings, such as downsizing the particle size [14,15], designing new morphologies [16,17], doping [18,19,20,21,22], carbon coating [23,24,25,26,27], and constructing hollow or porous structures [28,29,30,31,32,33].

Among all of these strategies, structuring porous MnO electrodes has been an effective strategy for enhancing the capacity retention by reversibly accommodating large volume changes. Additionally, the pores of the porous electrode provide good access for the electrolyte to the electrode surface. The large surface areas of the porous structures also facilitate charge transfer across the electrode/electrolyte interfaces [34]. 

Porous MnO materials can be synthesized by templated or non-templated methods, such as co-precipitation [35], hydrothermal, solvothermal [36], sol-gel, and deposition processes [37]. Among these synthesis methods, the co-precipitation method is simpler than the abovementioned processes and is suitable for large-scale production in commercial fields. Zhong et al. [35] synthesized porous MnO/C microspheres, which delivered a reversible capacity of 600 mAh·g^−1^ at a rate of 400 mA·g^−1^. These porous MnO particles were obtained with MnCO_3_ as the precursor through a co-precipitation method, and carbon was coated by chemical vapor deposition. However, the particle size and distribution from the co-precipitation method always depended on the mass transfer and dispersion in the reactor. In traditional co-precipitation, solutions are mixed by stirring for several hours [9,35], which does not easily control particle nucleation-growth processes. The micro-mixing intensity and mass transfer for the co-precipitation process need to be improved.

In this article, a novel and simple method for the fast synthesis of porous MnO/C microspheres with large specific areas is demonstrated for the first time. MnCO_3_ precursor was prepared in a T-shaped microchannel reactor in only a few seconds. Due to the enhancement of the mixing effect in the microchannel reactor, the calcinated production, MnO/C microspheres had a narrow size distribution and porous structure. These morphological and structural characteristics improved the electrochemical performance of MnO/C anodes [38,39].

## 2. Experimental

### 2.1. Preparation of Materials

**Figure 1:**
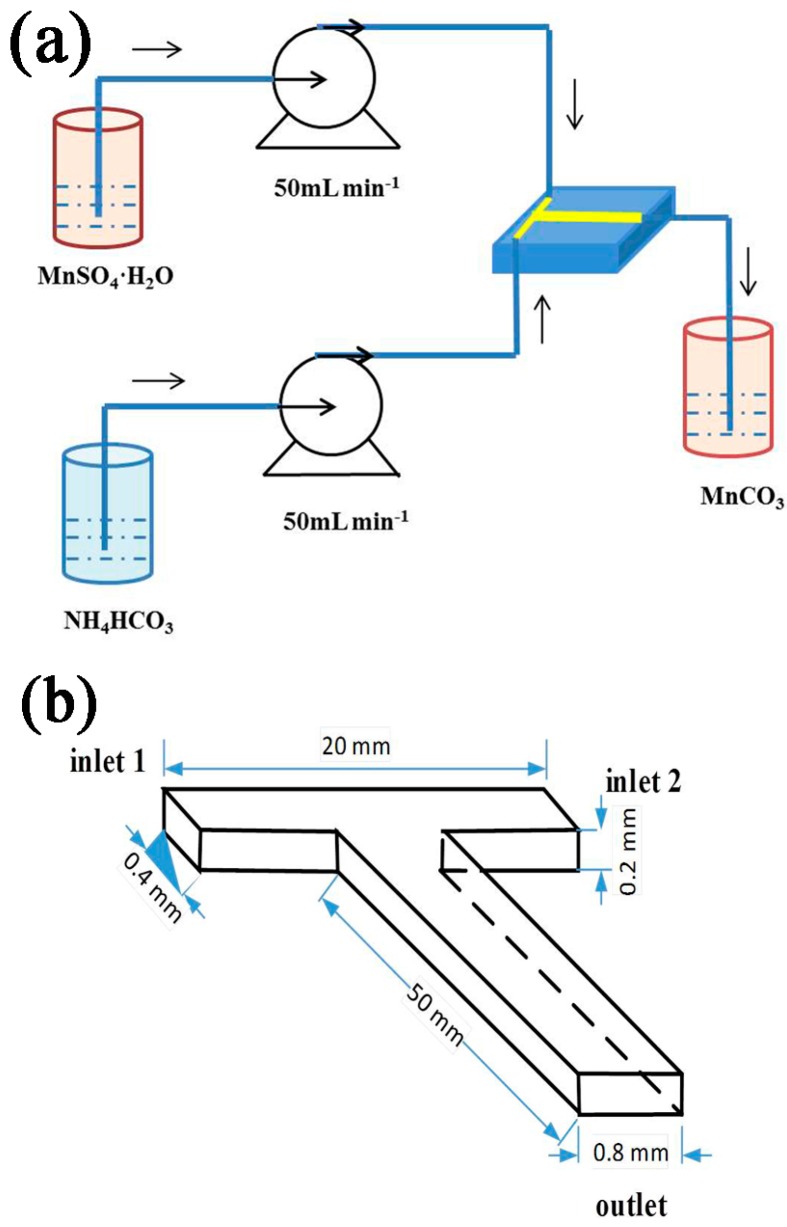
The experimental setup for the preparation of MnCO_3_ (**a**) and the sketch of the T-shaped microchannel reactor (**b**).

Porous MnO/C was prepared as follows: Two solutions of MnSO_4_·H_2_O (0.1 mol·L^−1^) and NH_4_HCO_3_ (0.2 mol·L^−1^) were simultaneously injected into the T-shaped microchannel reactor with an accurate syringe pump (2PB00C, Beijing Xingda Technology Co., Ltd., Beijing, China) at the same fixed flow rate of 50 mL·min^−1^ to generate the crystal MnCO_3_ (Figure 1a). The reaction mechanism of the MnCO_3_ preparation was as follows: MnSO_4_ + 2NH_4_HCO_3_ → MnCO_3_↓ + (NH_4_)_2_SO_4_ + CO_2_↑ + H_2_O.

Following this co-precipitation process, the product was collected by filtration, the sulfate ions were washed out (with the washing water tested with a 0.5 mol·L^−1^ BaCl_2_ solution), and dried in a vacuum oven at 60 °C. After that, the synthesized MnCO_3_ precursor was mixed with sucrose at a mass ratio of 10:3 by ultrasonication and dried at 60 °C. Finally, the composite was calcined in a tube furnace at 450 °C in N_2_ for 6 h to obtain porous MnO/C. During this calcination process, the MnCO_3_ precursor translated to MnO, and the CO_2_ gas released MnCO_3_ → MnO + CO_2_↑. 

The sketch of the T-shaped microchannel reactor is depicted in Figure 1b. The microchannel reactor has a mixing channel length of 50 mm, a width of 0.8 mm, and a depth of 0.2 mm, while each inlet channel has a width of 0.4 mm and a length of 10 mm. During the experiments, two opposite feed streams, MnSO_4_ and NH_4_HCO_3_ solutions, were mixed at the crunode, where the reactants impinge, then flowed through the vertical channel as the reaction proceeds.

### 2.2. Characterization of Materials

The crystalline structure and phase information of the as-prepared products were determined by X-ray powder diffraction (XRD) on an X’ Pert PRO X-ray diffractometer (D8 Advance, Bruker-AXS, Karlsruhe, Germany) with Cu Kα radiation (*V* = 40 KV, *I* = 40 mA and *λ* = 0.15406 nm) in the range of 10–80°. The morphology and particle sizes of the resultant samples were characterized with a field emission scanning electron microscope (FE-SEM, S-3400, Hitachi, Tokyo, Japan) and a transmission electron microscope (FE-TEM, Tecnai G2 F20, FEI, Hillsboro, OR, USA). The nitrogen adsorption–desorption isotherms were measured on a V-Sorb 2800 series analyzer (Gold APP Instruments, Beijing, China) to calculate the specific area by the Brunauer–Emmett–Teller (BET) analysis method and the pore size distribution by the Barrett–Joyner–Halenda method. The carbon content in the final product was tested using a Flash2000 elemental analyzer (Thermo Fisher Scientific, Waltham, MA, USA).

### 2.3. Electrochemical Measurements

The electrochemical performance of MnO/C was tested using CR2032 coin-type half cells. The working electrode was produced by mixing MnO/C, acetylene black, and a lithium polyacrylate (Li-PAA) binder [40] in a weight ratio of 8:1:1 in distilled water to form a homogenous slurry. The slurry mixture was coated on a copper foil substrate, followed by drying at 120 °C in a vacuum oven for 12 h. The loading of the active material in electrode plate is approximately 60 to 70 mg·cm^−2^. To set up the charge/discharge current and calculate the special capacity, the amount of loading of every electrode was weighted accurately. Half Li-ion battery cells were assembled in a glove-box filled with a dry and high-purity argon atmosphere. The coins use lithium metal foils as the counter/reference electrode, a Celgard 2300 as the separator, and 1 mol·L^−1^ LiPF_6_ (dissolved in ethylene carbonate and dimethyl carbonate with a volume ratio of 1:1) as the electrolyte. The galvanostatic charge and discharge measurements of the cells were evaluated on a NEWARE BTS Serier battery cycler (Neware, Shenzhen, Guangdong, China) at different current densities in a voltage range from 0.01 to 3 V with a precision of 0.05%. The charge/discharge current density and the specific capacity were calculated based on the whole weight of MnO/C in the electrode, where a 1 C rate was 755 mAh·g^−1^. Cyclic voltammogram (CV) measurements were conducted on a PCI 4750 electrochemical workstation (Gamry, Warminster, PA, USA) with a scan rate of 0.1 mV·s^−1^ and potential windows ranging from 0.01 to 3 V (versus Li/Li^+^). Electrochemical impedance spectroscopy (EIS) was completed using a Gamry PCI-4750 electrochemical workstation (Gamry, Warminster, PA, USA) in a frequency range from 100 kHz to 1 mHz.

## 3. Results and Discussion

### 3.1. Characterization of Samples

**Figure 2:**
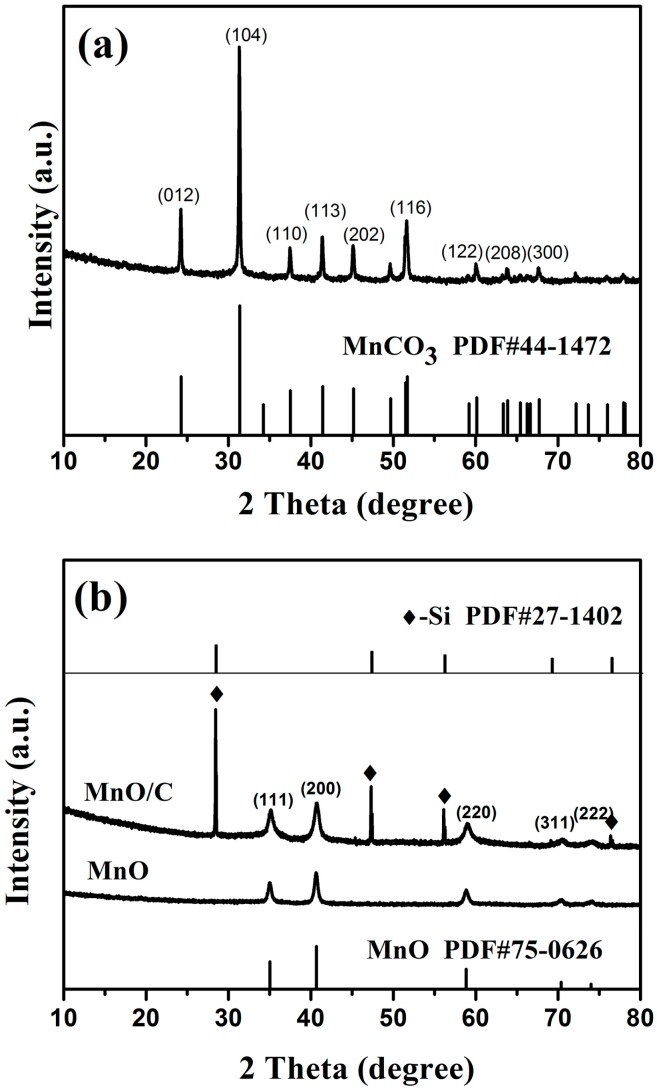
XRD patterns of MnCO_3_ precursor (**a**) and the calcined MnCO_3_ (**b**) (the top is the MnO/C sample; the bottom is the MnO sample).

The XRD patterns of the MnCO_3_ precursor are shown in Figure 2a. All diffraction peaks agree perfectly with the rhombohedral MnCO_3_ structure (JCPDS 44-1472), and no other phases were detected, indicating that the high-purity MnCO_3_ precursor had been prepared by the T-type microchannel reactor. The XRD characteristic peaks of the pure phase MnO and MnO/C composites are presented in Figure 2b. All of the peaks of the products were coincident with the standard XRD pattern of cubic MnO (JCPDS 75-0626), and no impure diffraction peaks were observed, confirming that MnCO_3_ had been completely decomposed into MnO without the generation of any other substance, and the carbon layer formed on the MnO surface was amorphous. The lattice constants of the MnO/C phase were calculated as *a* = *b* = *c* = 4.434(1) Å, *V* = 87.18 Å, and *α* = *β* = *γ* = 90°, which coincided well with the literature [41,42,43].

**Figure 3:**
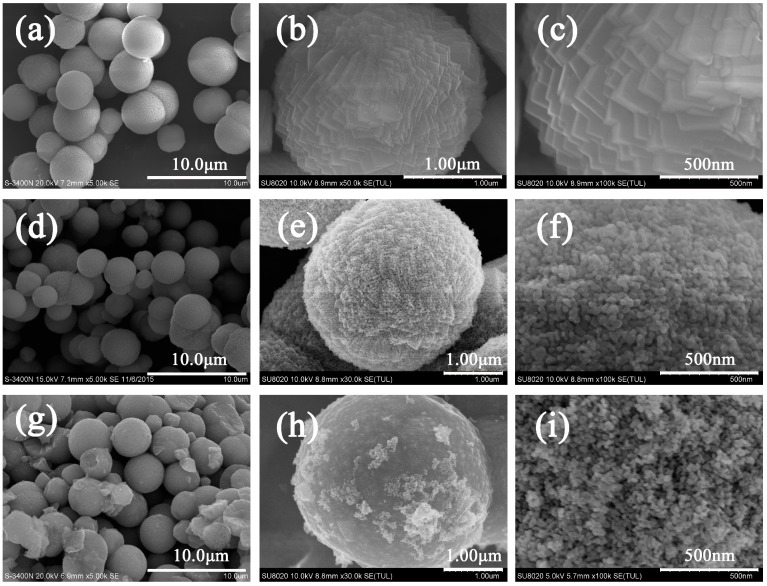
FE-SEM images of MnCO_3_ (**a**–**c**), MnO (**d**–**f**), and MnO/C (**g**–**i**).

Figure 3a–c present the SEM images of the prepared MnCO_3_ microspheres with approximate sizes distributions of 2–3 μm, and the surface of the microspheres consist of flake-like particles with a thickness of about 50 nm. Compared to MnCO_3_ prepared in bulk batch reactors [35], our microchannel reactor can develop MnCO_3_ with a smaller mean particle size mainly due to a better mixing effect and improved mass transfer performance in the microfluidic device [44]. The Reynolds number for the feed channel was 2781 when the flow rate was 50 mL·min^−1^ (H_2_O in 10 °C was used as the flow media), which is much larger than the 1110 that was reported for a similar microchannel reactor [45]. The impinging of liquid with a high velocity and a large Reynolds number at the T-junction induced a high intensity vortex, which led to the efficient micro-mixing and preparation of the MnCO_3_ microspheres.

The SEM images in Figure 3d,g demonstrated the morphological characteristics of MnO and MnO/C, with no apparent variations of the spherical structure during the calcination process. Figure 3e,f,h,i show that the surface of MnO/C is glossier than MnO, on account of the formation of the carbon stratum. For the inner morphology of MnO, the microspheres were composed of loosely packed primary particles with diameters of about 50 nm and tiny porous structures that were generated by CO_2_ release during calcination. However, the emancipating CO_2_ also caused a volume expansion that led to an increase in the particle sizes of MnO and MnO/C, which had proximate diameters of 3–4 μm.

**Figure 4:**
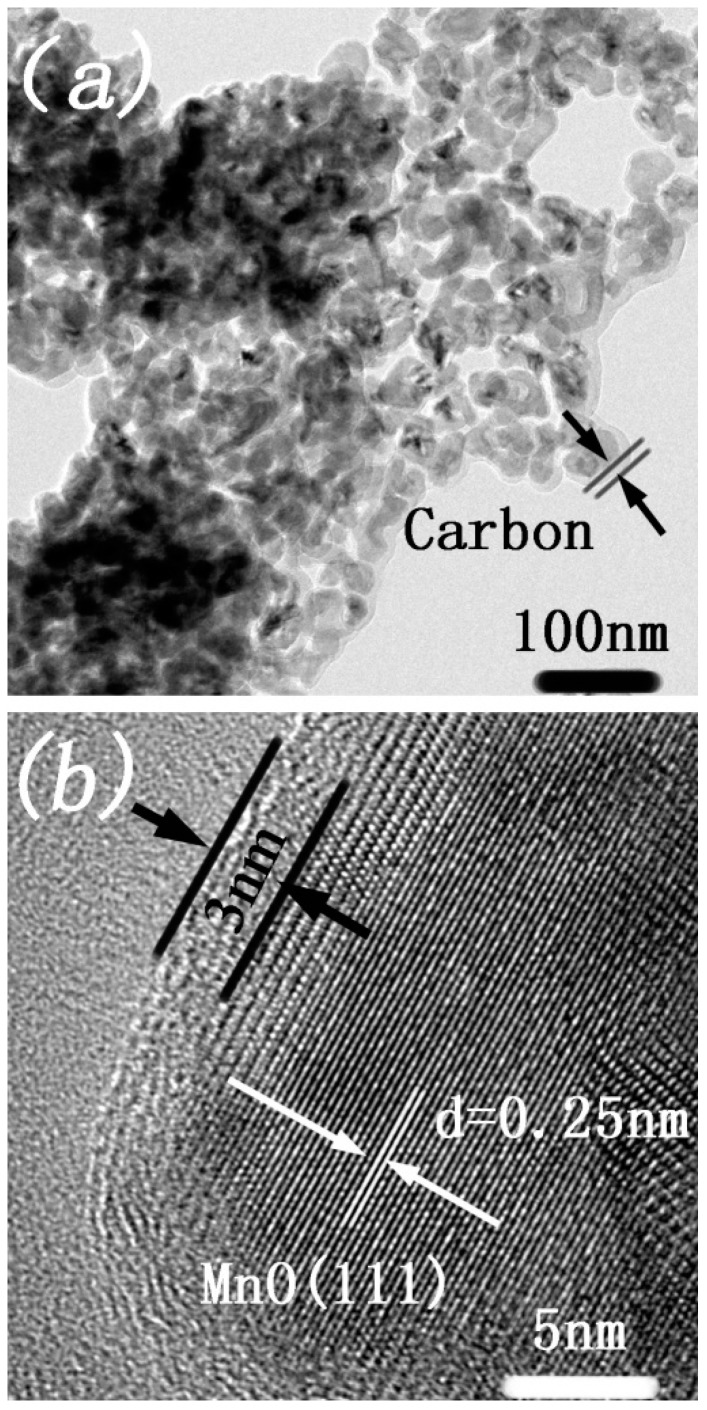
FE-TEM images of MnO/C microspheres (**a**) and the carbon layer (**b**).

The FE-TEM images of the MnO/C microspheres are exhibited in Figure 4a. The prepared MnO/C microspheres were composed of nano-sized grains, and the voids between the nanoparticles form a porous framework. The HRTEM image in Figure 4b shows a continuous and uniform carbon layer (about 3–4 nm) along the MnO surface and the carbon content of the final sample was 21.9 ± 0.3%. This clearly demonstrated that the lattice fringe spacing is about 0.25 nm, corresponding to the cubic MnO XRD results for the (111) crystal plane.

**Figure 5:**
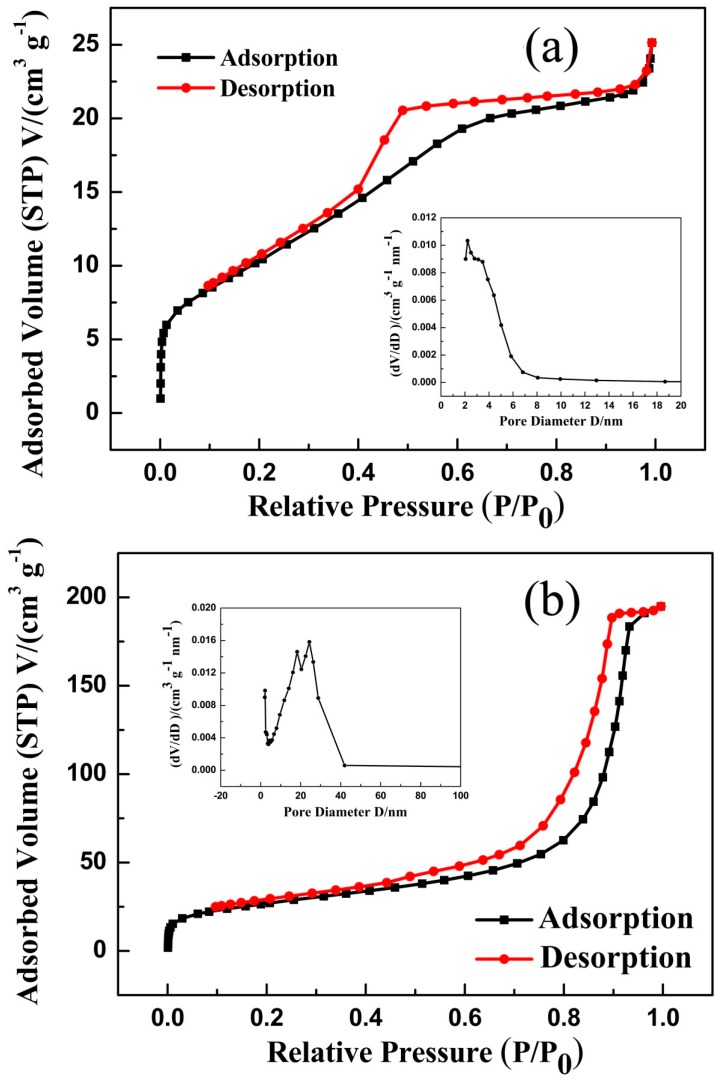
Nitrogen adsorption–desorption isotherms of MnCO_3_ (**a**) and MnO/C (**b**). The insets show the two samples of the pore-size distributions.

The BET specific surface areas and porous structures of MnCO_3_ and MnO/C were further measured by nitrogen adsorption–desorption isotherms. Figure 5 shows the isotherms and pore size distribution of the two specimens. The isotherm of MnCO_3_ exhibits type IV isotherm behavior with obvious H2 hysteresis loops and MnO/C displays typical IV isotherms with H3 hysteresis loops, both of the samples confirm the presence of mesopores. From the inset of Figure 5a, the average pore size of MnCO_3_ is 2.22 ± 0.01 nm and the BET surface area is measured to be 38.06 m^2^·g^−1^ with a standard deviation of 1%. The corresponding pore size distribution of MnO/C is clarified in the inset of Figure 5b, with an average pore size of 24.37 ± 0.01 nm in diameter, in accordance with the FE-SEM and TEM images, and a BET specific surface area of 96.66 m^2^·g^−1^. The BET surface area and the pore size of MnO/C are larger than MnCO_3_ is mainly attributed to the release of CO_2_ during the decomposition of MnCO_3_. In addition, such a porous structure, with a specific surface area higher than that found in previous studies [31,41,46,47], can not only accelerate the diffusion of Li^+^ between the electrolyte and internal active materials but also buffer the volume variation during Li^+^ insertion/extraction to promote the reversible capacity and the stability of the cycling performance [34,46,48].

### 3.2. Electrochemical Performance of the MnO/C Electrode

**Figure 6:**
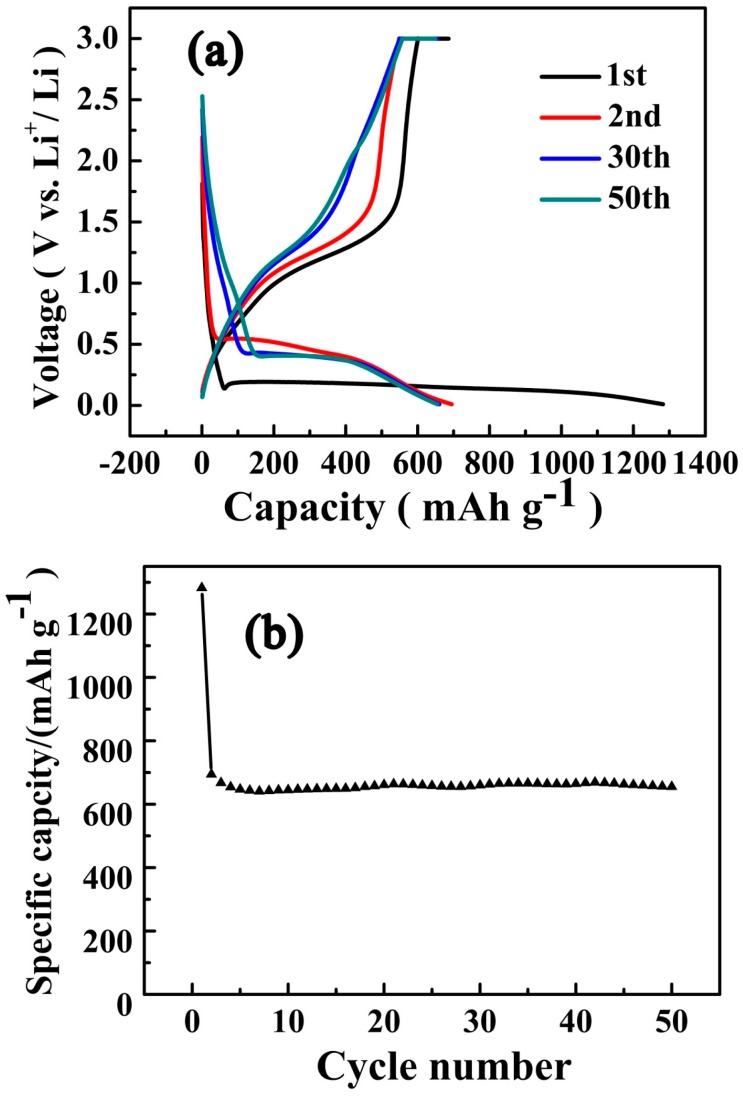
Charge–discharge curves (**a**) and cycling performance (**b**) of the prepared MnO/C sample at a rate of 1 C.

To examine the electrochemical storage properties of the as-prepared porous MnO/C material, galvanostatic discharge–charge cycling was firstly explored, as shown in Figure 6. The initial and second discharge-specific capacities of the MnO/C sample at 1 C (755 mA·g^−1^) were 1282.3 and 694.4 mAh·g^−1^, respectively. The first discharge capacity was much higher than the theoretical value (755 mAh·g^−1^). The irreversible capacity loss in the first cycle was mainly attributed to the formation of the solid electrolyte interface (SEI) layer and, to a certain extent, decomposition of the electrolyte [33,36,49,50]. However, from the second cycle, the MnO/C electrodes exhibit excellent cycling performance, accompanied with coulombic efficiency, which was maintained at almost 100%. The first discharge voltage plateau at 0.1 V in Figure 6a corresponded to the initial reduction of MnO to Mn and the formation of the SEI layer [23,33,51], then the discharge plateau turned to 0.3 V in the subsequent cycles, indicating the irreversible phase transformation, owing to the formation of Li_2_O and metallic Mn [7,33,47,52]. The charge curves show no voltage plateau, but a slope from 1.0 to 1.5 V, suggesting the oxidation of Mn to MnO [13,15,47]. From Figure 6b, we discover that the electrolyte decomposition did not happen from the second cycle, and the discharge-specific capacity after 50 cycles can still be retained as high as 654.8 mAh·g^−1^ with a capacity retention rate of 98%, except for the initial two cycles, thus exhibiting excellent electrochemical properties compared with the MnO/C prepared by the traditional co-precipitation method [22,35]. 

**Figure 7:**
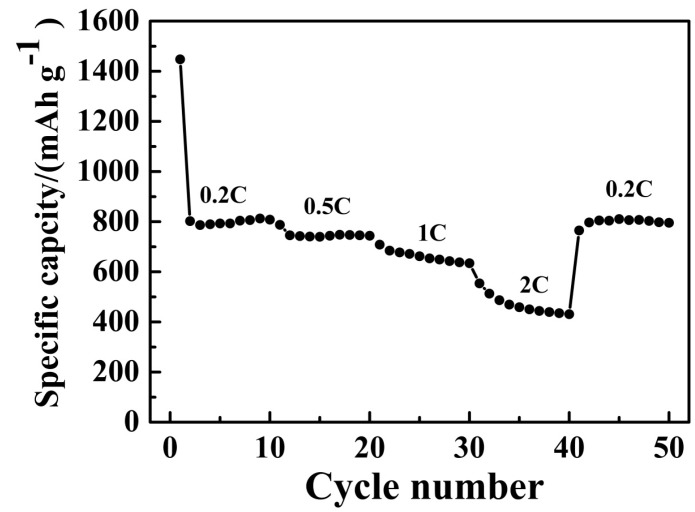
Rate performances of the porous MnO/C at various current rates.

The rate capacities of the porous MnO/C microspheres at various current densities are exhibited in Figure 7. The corresponding discharge capacities reach 808.3, 743.7, 642.6, 450.1, and 803.1 mAh·g^−1^ at 0.2, 0.5, 1, 2, and 0.2 C, respectively. It is worth noting that when the current density was decreased back to 0.2 C, the discharge capacity of 803.1 mAh·g^−1^ was almost 100% recovered, illustrating the excellent rate capability. The outstanding electrochemical performance of MnO/C might be attributed to the porous structure with a high specific surface area and the carbon coating, which can effectively accommodate the stress and strain of the volume change and hinder the agglomeration and separation of MnO during the Li-ion insertion/extraction process [33]. In comparison with the traditional co-precipitation method [9,22,23,24,30,35], our method not only presents a sample with remarkable specific capacity, significant cycling stability, and excellent rate performance, but also shortens the reaction time, strengthens the mixing effect, and makes it easy to control the reaction process. Clearly, these results indicate that our T-shaped microchannel reactor method is a very promising method for synthesizing high-performance MnO/C and related materials. 

**Figure 8:**
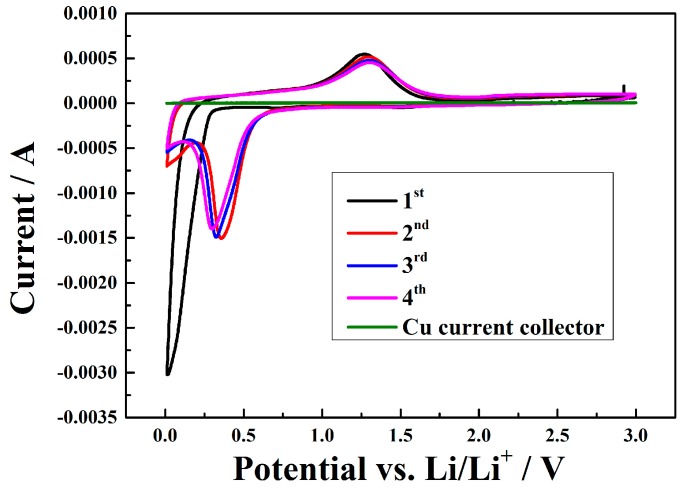
CV curves of the porous MnO/C and Cu current collector at a scan rate of 0.1 mV·s^−1^.

The CV curves of the porous MnO/C composite electrode in a voltage range from 0.01–3.0 V with a scan rate of 0.1 mV·s^−1^ for the first four cycles are shown in Figure 8. The CV curve for Cu current collector, which is without any oxidation or reduction peak in the above voltage range is also shown in this figure. In the first cathodic sweep for MnO/C composite electrode, only one sharp reduction peak close to 0.1 V is observed, agreeing well with the shaping of the SEI layers and the reduction of Mn^2+^ to Mn^0^. From the second cycle onwards, the main reduction current peak turns to 0.3 V, suggesting the formation of Li_2_O and metallic Mn, presented as MnO + 2 Li → Mn^0^ + Li_2_O, which is an irreversible phase transformation [3,28]. In the oxidation half cycle, a wide main peak is observed at 1.3 V, in good accord with the oxidation of Mn^0^ to Mn^2+^ and the decomposition of Li_2_O [30,37,53]. Both the reduction and oxidation curves in the subsequent cycles overlap well, demonstrating the excellent reversibility of the electrochemical reaction, which matches well with the charge/discharge experiments. 

**Figure 9:**
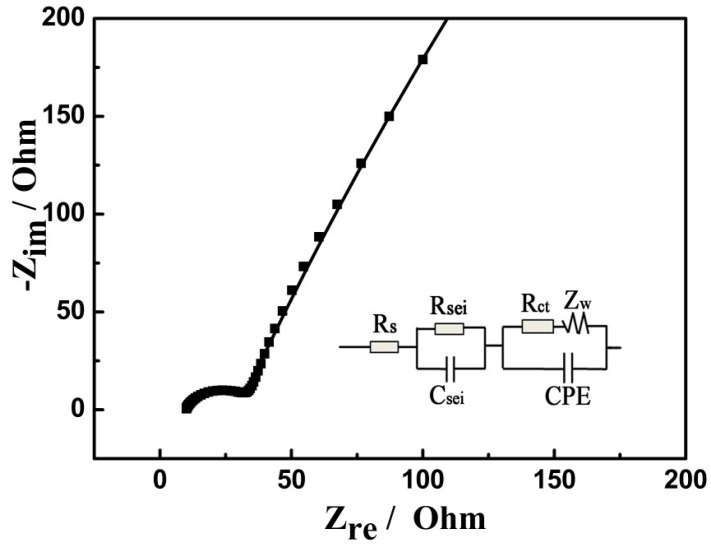
Impendence spectra for the prepared porous MnO/C.

EIS was further executed to explore the electrochemical characteristics of the electrode/electrolyte interface. Figure 9 shows the Nyquist plot of the MnO/C electrode without any discharge/charge cycles, and the inset gives an equivalent circuit of the impedance spectra. The intercept of the high-frequency semicircle on the *Z’* axis is ascribed to the resistance of the electrolyte (*R_s_*). The high-frequency semicircle is associated with the capacitance (*C*_sei_) and the resistance (*R*_sei_) of the SEI layer. The middle-frequency semicircle is attributed to the charge transfer resistance (*R*_ct_) between the electrodes. The straight line in the low frequency is consistent with Warburg impedance (*Z*_w_), corresponding to the diffusion of Li-ions into the bulk electrode [33,42]. The fitting values of the kinetic parameters of the MnO/C electrode are listed in Table 1. It is worth noting that *R*_ct_ is much lower than that previously reported [13,22], indicating much higher reaction areas and a faster charge transfer at the electrode/electrolyte interface [9,33], which is attributed to the high surface area of the mesoporous structure and the carbon coating that facilitates the charge–transfer reaction. The diffusion coefficient of lithium ions (*D*_Li_) in the MnO/C sample is 3.64 × 10^−18^ cm^2^·s^−1^, which is larger than that reported by Yang et al. [18]. These results further illustrate that the porous MnO/C microspheres should display excellent electrochemistry performance.

## 4. Conclusions

In this work, we demonstrated a novel, economical, and facile strategy for fabricating porous MnO/C microspheres by employing a T-shaped microchannel reactor. The resulting porous MnO/C microspheres had an average pore size of 24.37 nm and a larger specific surface area of 96.66 m^2^·g^−1^. As anode materials for Li-ion batteries, the prepared porous MnO/C microspheres demonstrated electrochemical performance with a discharge capacity of 655.4 mAh·g^−1^ at 1 C after 50 cycles, which is mainly ascribed to the mesoporous structure and the carbon coating that stimulated Li-ion diffusion into the cathode by increasing the electrochemical reaction surface. The approach used in this work provides a fast, easily controllable, and mass-producible method of fabricating porous MnO/C composites, and can dramatically save time and cost.

## Figures and Tables

**Table 1:** Simulation results of EIS in Figure 9.

*R*_s_/Ω	*C*_sei_/F·cm^−2^	*R*_sei_/Ω	CPE *Y*_o_/S·s^n^·cm^−2^	*R*_ct_/Ω	*Z*_w_/S·s^0.5^·cm^−2^
3.88	6.45 × 10^−5^	23.62	3.36 × 10^−4^	16.32	0.00889
